# Is hyperuricemia an independent risk factor for new-onset chronic kidney disease?: a systematic review and meta-analysis based on observational cohort studies

**DOI:** 10.1186/1471-2369-15-122

**Published:** 2014-07-27

**Authors:** Ling Li, Chen Yang, Yuliang Zhao, Xiaoxi Zeng, Fang Liu, Ping Fu

**Affiliations:** 1 Division of Nephrology, West China Hospital of Sichuan University, Chengdu 610041, Sichuan, China; 2 Division of General Surgery, West China Hospital of Sichuan University, Chengdu 610041, Sichuan, China

**Keywords:** Chronic kidney disease, Hyperuricemia, Uric acid

## Abstract

**Background:**

Hyperuricemia has been reported to be associated with chronic kidney disease (CKD). However whether an elevated serum uric acid level is an independent risk factor for new-onset CKD remained controversial.

**Methods:**

A systematic review and meta-analysis using a literature search of online databases including PubMed, Embase, Ovid and ISI Web/Web of Science was conducted. Summary adjusted odds ratios with corresponding 95% confidence intervals (95% CI) were calculated to evaluate the risk estimates of hyperuricemia for new-onset CKD.

**Results:**

Thirteen studies containing 190,718 participants were included. A significant positive association was found between elevated serum uric acid levels and new-onset CKD at follow-up (summary OR, 1.15; 95% CI, 1.05–1.25). Hyperuricemia was found be an independent predictor for the development of newly diagnosed CKD in non-CKD patients (summary OR, 2.35; 95% CI, 1.59–3.46). This association increased with increasing length of follow-up. No significant differences were found for risk estimates of the associations between elevated serum uric acid levels and developing CKD between males and females.

**Conclusions:**

With long-term follow-up of non-CKD individuals, elevated serum uric acid levels showed an increased risk for the development of chronic renal dysfunction.

## Background

The prevalence of chronic kidney disease (CKD) is increasing in various populations worldwide, and risk factors associated with CKD have gained much attention in recent years [1]. Hypertension, lifestyle and metabolic syndrome among other factors have been associated with the incidence of CKD in previous studies [2-4]. Since the 1990s, nephrologists have reported that hyperuricemia is associated with CKD. However, other risk factors such as hypertension, metabolic syndrome and gout have also been proven to be associated with hyperuricemia [5]. Therefore, whether an elevated uric acid level is an independent risk factor for CKD remains to be elucidated. Increasing evidence suggests that hyperuricemia may be a pathogenic factor for the development of CKD rather than just a marker of decreased renal uric acid excretion. Several potential mechanisms have been suggested to explain the causal relationship between hyperuricemia and CKD, including vascular smooth cell proliferation [6], endothelial dysfunction [7], increased synthesis of interleukin-6, impaired endothelial nitric oxide production [8] and insulin resistance [9]. In the past 3 years, etiologic studies have explored relationships between hyperuricemia and renal disease, and most studies revealed a positive association between elevated uric acid levels and CKD or new-onset CKD [10]. Some nephrologists recommend management of uric acid in CKD patients or groups at high risk of CKD [11].

The current meta-analysis was performed to quantitatively assess relationships between hyperuricemia and CKD, and also to evaluate whether associations were influenced by hypertension, hyperuricemia or other confounding factors, and whether they varied by study design, sex and geographical area.

## Methods

### Study selection

A literature search was performed using electronic databases Medline Ovid/Medline (1948 to present), PubMed/Medline, Embase, and ISI Web/Web of Science for published studies from January 1970 to September 2013. Key words used were: “hyperuricemia”, “uric acid elevated”, “hyperuricaemia”, “uricacidemia”, and “uricacidaemia”; and “CKD” or “chronic kidney disease” or “chronic renal dysfunction” or “chronic renal insufficiency”. The “related items” function was used to broaden the search. The electronic search was up to September 2013 with no limitations regarding the type of publication. Studies were screened by two reviewers (L.L. and C.Y.) independently with the following inclusion criteria: (1) report of the association between serum uric acid (SUA) and new-onset CKD; (2) studies of observational cohort design; and (3) report of the hazard ratio (HR) or odds ratio (OR) in cohort studies with 95% confidence intervals (95% CI) or sufficient information to calculate these figures. Exclusion criteria were: (1) studies published in languages other than English; and (2) studies reporting on patients with end-stage renal disease or acute renal impairment or patients requiring dialysis. The definition of CKD was: eGFR <60 mL/min per 1.73 m^2^ for more than 3 months or the presence of albuminuria (kidney damage) for more than 3 months, according to the National Kidney Foundation Kidney Disease Outcomes and Quality Initiative (KDOQI) CKD guidelines [12]. Studies that used other definitions for CKD, such as serum creatinine levels, were excluded. If more than one study reported duplicated material, the most recent study was included in the systematic review. Reference lists of all included studies were reviewed for any additional relevant studies. The whole process of this systematic review was conducted in strict accordance with the criteria of the moose checklist to ensure the quality of this study (Additional file 1).

### Outcome measure

KDOQI CKD guidelines were used for the definition of CKD severity: Stage 1, GFR >90 mL/min per 1.73 m^2^ with microalbuminuria or macroalbuminuria; Stage 2, GFR 60–89 mL/min per 1.73 m^2^ with microalbuminuria or macroalbuminuria; Stage 3, GFR 30–59 mL/min per 1.73 m^2^; and Stage 4 GFR 15–29 mL/min per 1.73 m^2^[12]. Two reviewers (L.L. and C.Y.) retrieved the following data from the included studies independently: author, year published, country where study was performed, material source, cut-off level for hyperuricemia, sample size (number of patients and controls), SUA levels in CKD and non-CKD groups (mean ± SD), variables adjusted for calculation in the analysis, and adjusted OR or HR estimates with corresponding 95% CI. If the study classified participants according to quartiles of uric acid level, the highest quartile was taken to indicate hyperuricemia. Data reporting on the OR or HR of hyperuricemia and new-onset CKD or CKD risk were retrieved and combined.

### Quality assessment

The Newcastle–Ottawa Scale (NOS) was used for meta-analysis of observational studies in epidemiology for assessment of quality [13]. Risk of bias was assessed among the cohort studies on three aspects: (1) selection of participants (containing four domains); (2) comparability (one domain); and (3) outcome measure (containing three domains). Each domain was rated as “Yes”, “No”, or “Unclear”. Each quality domain was categorized as low risk for bias (Yes) when adequate data were reported to assess the study quality or the study meeting the criteria, and high risk (No) when the study reported adequate data but did not meet the criteria for that quality domain. Studies that did not report data to assess quality were categorized as “Unclear” and thus potentially at high risk of bias. “Yes” was scored 1 and “No” or “Unclear” were scored “0”. A quality bar was plotted for each domain to examine the limitations of the studies. Studies of high quality were defined as score >5 points.

### Statistical analysis

Statistical analysis was performed in accordance with The Cochrane Library handbook and the Quality of Reporting of Meta-Analyses guidelines [14,15]. Different measures for risk estimates from multiply analyses, such as HR (Cox regression) or OR (logistic regression), were extracted from studies; only the most adjusted risk estimates that examined associations between SUA level or hyperuricemia and new-onset CKD were extracted and pooled. We used the random effects model for pooled analysis of adjusted odds ratios (AOR) because of anticipated statistical heterogeneity, but the fixed effects model was also used to ensure robustness of the model chosen and susceptibility to outliers. Heterogeneity among studies was assessed using the *χ*^2^ test. *P-*values <0.05 were considered statistically significant.

Meta-regression analysis was conducted to explore heterogeneity among studies. Possible sources of heterogeneity included participant-specific characteristics such as age, sex, location, and source of participants; and study-specific characteristics such as data type for calculating HR or OR (continuous variables or dichotomous variables), sample size, study quality, and definitions used for hyperuricemia. Funnel plots and Egger regression tests were used to investigate publication bias. We used RevMan version 5.1 (The Cochrane Collaboration, The Nordic Cochrane Centre, Copenhagen, Denmark) and Stata version 11.0 for Windows (Stata Corp., College Station, TX, USA) for data analysis and graph formation.

## Results

### Search results

**Figure 1:**
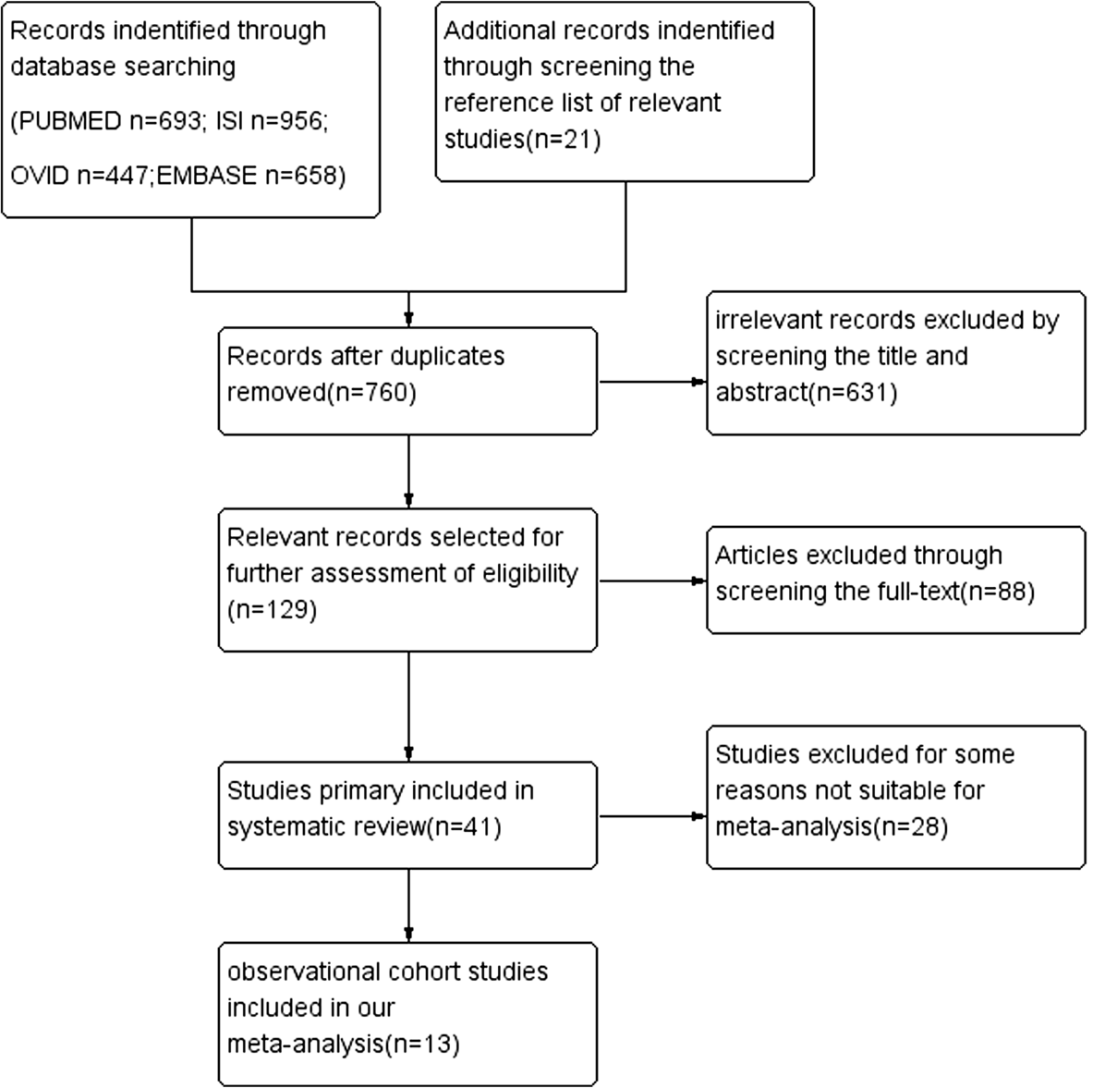
Study flow diagram of study selection.

We identified 1662 relevant citations in the combined search of online electronic databases (partial overlap) and 21 additional citations from the reference lists of relevant articles. After removing duplicated records, 790 studies were screened by examining titles and abstracts, and 129 relevant studies after primary selection were reviewed by reading the complete text for eligibility. There were 41 studies that reported on associations between SUA and renal disease, and these were assessed in detail. Articles with a cross-sectional or case–control style, a different definition of CKD or insufficient data were excluded. Finally, 13 studies that met inclusion criteria were included in our meta-analysis (Figure 1).

### Study characteristics

The characteristics of the 13 studies are detailed in Table 1. A total of 190,718 participants were included, with sample sizes ranging from 324 to 94,422. Four studies included the United States or populations of Western countries, and nine studies were conducted in Asian populations. Nine studies reported the relationship between increasing SUA level and the development of new-onset CKD during follow-up of non-CKD participants (AOR calculated on continuous variables per 1 mg/dL), and seven studies presented risk estimates for associations between hyperuricemia and the incidence of CKD (AOR calculated on dichotomous variables). The NOS quality assessment of the included cohort studies is listed in the Additional file 2. Eight cohort studies showed a comparatively high quality (NOS >5).

**Table 1:** Characteristics of studies reporting the relationship between hyperuricemia and CKD

**Author/year**	**Country**	**Study type**	**Follow-up (mean)**	**Source**	**Selective criteria for hyperuricaemia**	**Defination of CKD**	**Events**		**AOR (AHR) (95% CI)**	**Adjustments**
							**HPU**	**Control**		
**Ryoo *et al. *** **[**[16]**] 2013**	Korea	Prospective cohort	4 years	CP	>7.0 mg/dL	eGFR < 60	44/3910	66/14868	1.96 (1.28–2.99)^#^	Age, BMI, Diabetes, smoking, alcohol, blood pressure, exercise, hyperlipidaemia
**Chang *et al. *** **[**[17]**] 2013**	Taiwan	Prospective cohort study	4 years (>40 years old)	CP	>7.5 mg/dL (male) and >6.5 mg/dL (female)	ACR > 30	N.R.	N.R.	1.42 (1.27-1.59)&	Age, gender, BMI, Diabetes, blood pressure, Hypercholesteraemia, education
									3.54 (2.11,5.94)^#^	
**Zoppini *et al. *** **[**[18]**] 2012**	Italy	Retrospective cohort	5 years	DP	>7.0 mg/dL (male); >6.5 mg/dL (female)	eGFR < 60 or macro-albuminuria	47/159	147/1290	1.20 (1.03–1.57)& 2.01	BMI, smoking, blood pressure, albuminuria, duration of diabetes, HbA1c
									(1.10–3.74)^#^	
**Sonoda *et al. *** **[**[19]**] 2011**	Japan	Prospective cohort	4–5 years	CP	>7.0 mg/dL (male); >6.0 mg/dL (female)	eGFR < 60	N.R.	N.R.	1.09 (1.01–1.18)&	BMI, BP, LDL, HDL, smoke, eGFR
**Kawashima *et al. *** **[**[20]**] 2011**	Japan	Retrospective cohort	95.2	CP	>7.0 mg/dL	eGFR < 60	32/166	68/1119	3.99 (2.59–6.15)^#^	Age, BMI, HDL, BP, blood
			(±66.7) months							
**Mok *et al. *** **[**[21]**] 2011**	Korea	Severance cohort	6.5 years	CP	>6.6 mg/dL (male); >4.6 mg/dL (female)	GFR <60	226/3450	540/11489	2.1 (1.6–2.9) male*;	BMI, Diabetes, blood pressure, Hypercholesteraemia
									1.3 (1.0-1.8) female*	
**Yamada *et al. *** **[**[22]**] 2011**	Japan	Retrospective cohort	5 years	CP	>6.7 mg/dL (male); >4.8 mg/dL (female)	eGFR <60	343/3119	282/11280	1.42 (1.28–1.58) male&;	Age, BMI, Diabetes, smoking, alcohol, blood pressure, albuminuria, hyperlipidaemia
									1.32 (1.12–1.56) female&	
**Wang *et al. *** **[**[23]**] 2011**	China	Prospective cohort	3 years	CP	>7.0 mg/dL (male); >6.0 mg/dL (female)	GFR <60	N.R.	N.R.	1.03 (1.01–1.06)&	Age, gender, BMI, smoking, alcohol, exercise, Hypercholesteraemia, education, hyperlipidaemia
**Jalal *et al. *** **[**[24]**] 2010**	America	Prospective observational study.	6 years	DP	N.R.	ACR > 30	N.R.	N.R.	1.80 (1.20-2.80)^#^	Age, gender, BMI, blood pressure, albuminuria, duration of diabetes, HbA1c, serum creatinine, medication for CKD or hyperuricaemia
**Yen *et al. *** **[**[25]**] 2009**	Taiwan	Prospective cohort	32.4 months	CP	> 6.6 mg/dL	eGFR < 60	84/312	60/488	0.997 (0.847–1.175)&	Age, gender, BMI, Diabetes, smoking, blood pressure, Hypercholesteraemia, albuminuria, serum creatinine
				(>65 years old)						
**Weiner *et al. *** **[**[26]**] 2008**	America	Prospective cohort	8.5 years	CP	>7.4 mg/dL (male); >6.1 mg/dl (female)	eGFR < 60	260/3167	481/10171	1.07 (1.01–1.14)&	Age, gender, race, diabetes, BP, cardiac disease, smoke, alcohol use, education, lipid, albumin
**Obermayr *et al. *** **[**[27]**] 2008**	Austria	Retrospective cohort	7 years	CP	7.0–8.9 mg/dL	eGFR < 60	N.R.	N.R.	1.26 (1.02–1.55)&	Age, gender, Diabetes, LDL, hyperlipidaemia, medication for CKD or hyperuricaemia
**Domrongkitchaiporn *et al. *** **[**[28]**] 2005**	Thailand	Retrospective cohort	12 years	CP	6.30–14.50 mg/dL	eGFR <60	N.R.	N.R.	1.82 (1.12–2.98)^#^	BMI, Diabetes, smoking, blood pressure, Hypercholesteraemia, albuminuria

&AOR calculated using uric acid level for incidence of onset of CKD; ^#^AOR calculated using hyperuricemia individuals compared with normal individuals for new-onset CKD; *AOR calculated using individuals of the last uric acid quartiles compared with first uric acid quartiles for the risk for CKD; N.R.: not reported; eGFR <60 means eGFR <60 mL/min/1.73 m^2^; ACR >30 means urinary albumin-to-creatinine ratio >30 mg/g; HPU: hyperuricemia; CP: community-based population; DP: patients with diabetes mellitus.

The main limitation observed in the majority of studies was unclear reporting of dropout rates, as many studies did not mention uncompleted follow-ups.

Four studies provided the AOR for uric acid level as a risk factor for the development of CKD in males and females separately. Three studies grouped participants in quartiles according to uric acid level (the OR calculated for the highest quartile compared with the lowest quartile as a reference was retrieved for pooled analysis). Eleven studies defined CKD as the development of eGFR <60 mL/min per 1.73 m^2^, while the remaining two used albuminuria for the definition.

With regard to choice of participants, 11 studies used population-based participants who underwent health examinations, while the source in the other two studies was hospital-based participants with diabetes mellitus [18,24].

Different cut-off levels were used for defining hyperuricemia (Table 1). An SUA level >7.0 mg/dL in men or >6.0 mg/dL in women was used in the majority of studies.

Adjustments made for potential confounders of at least three factors were mentioned in all 13 studies.

### Serum uric acid level and development of new-onset CKD

**Figure 2:**
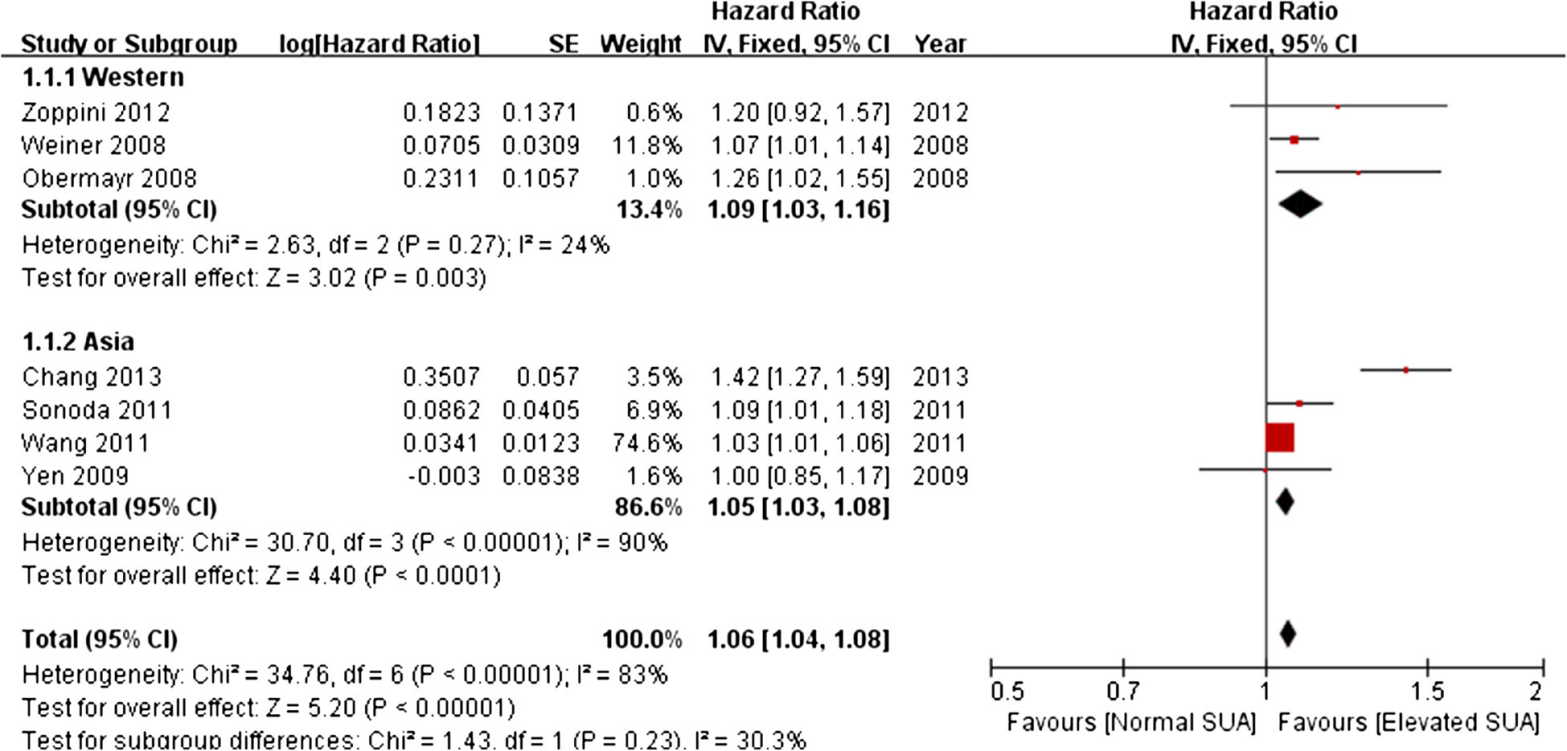
Forest plot of association between SUA and new-onset CKD.

Seven studies provided the adjusted OR of increasing risk of new-onset CKD per 1 mg/dL SUA elevated. The results of pooled analysis revealed a significant positive association between SUA and incidence of CKD (summary OR, 1.06; 95% CI, 1.04–1.08) with evidence of significant heterogeneity among studies (*Q* = 34.76, *P* < 0.001, *I*^2^ = 83%) (Figure 2).

**Figure 3:**
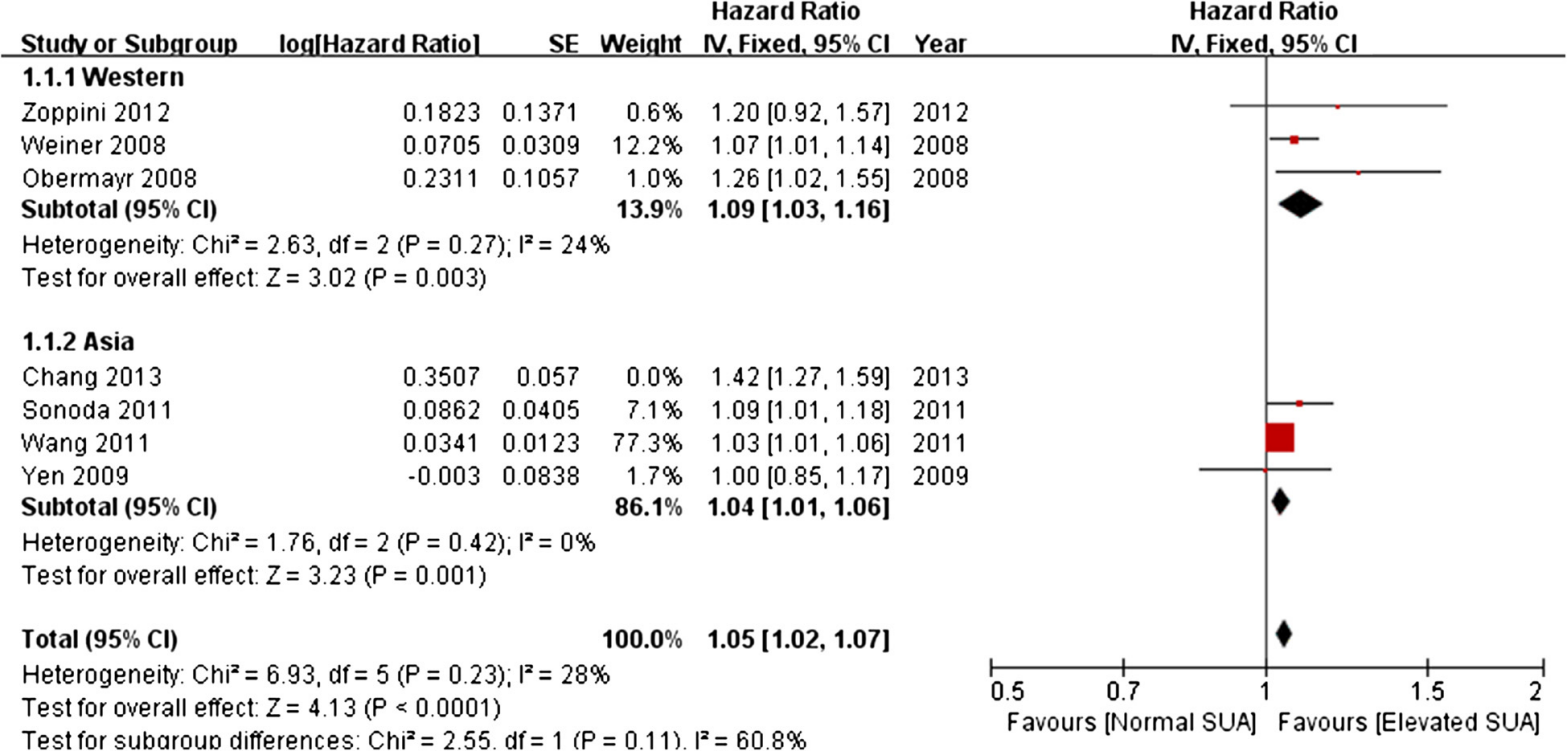
**Forest plot of association between SUA and new-onset CKD (excluding the study by Chang et al. [**[17]**]).**

On examining study variability using meta-regression analysis, we found that the population age was the cause of heterogeneity. In one study that used an elderly population [17], the OR was comparatively higher than the other studies. By excluding this particular study, we observed that a clear positive association was retained (summary OR, 1.05; 95% CI, 1.02–1.07) while heterogeneity was greatly reduced (*Q* = 6.93, *P* = 0.23, *I*^2^ = 28%) (Figure 3).

### Hyperuricemia and development of new-onset CKD

**Figure 4:**
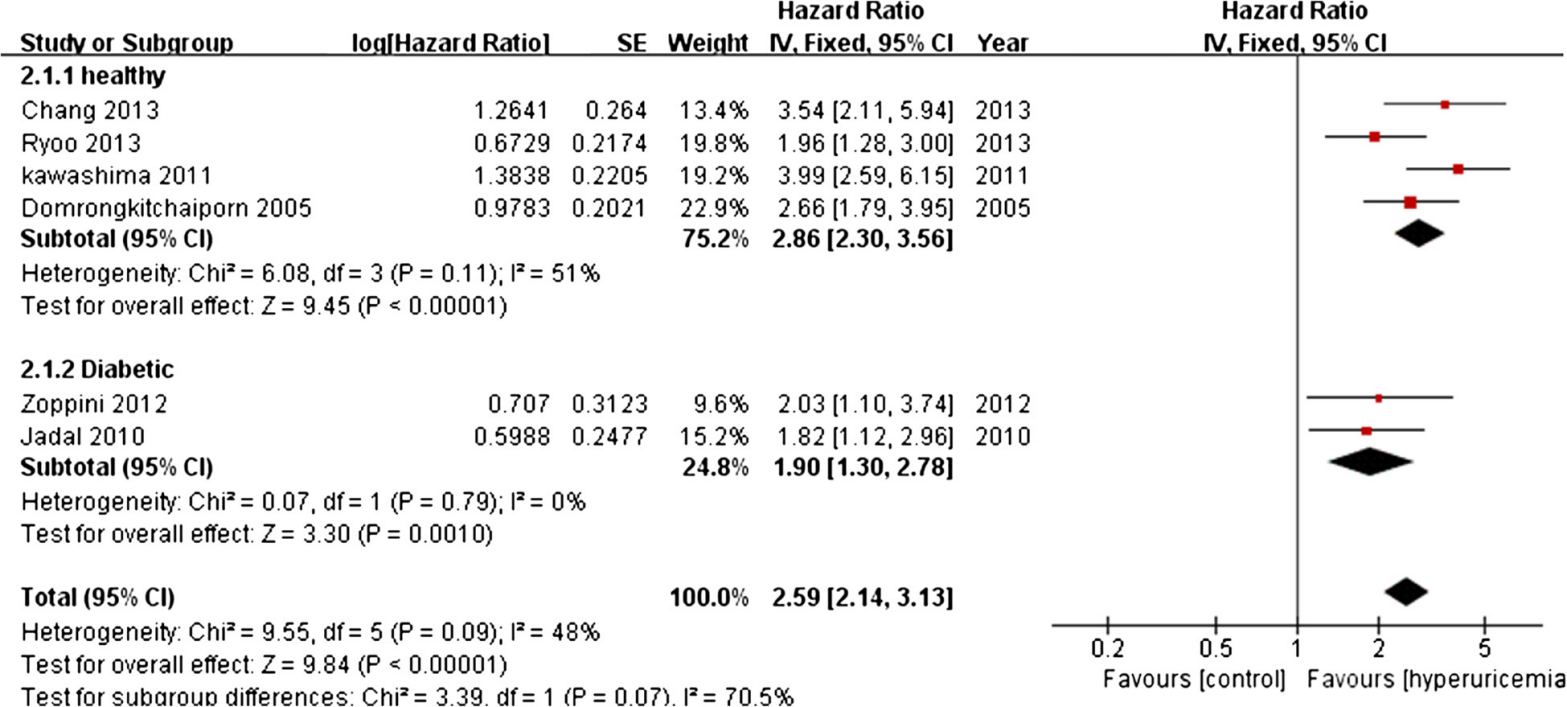
Forest plot of association between hyperuricemia and new-onset CKD.

Six studies provided risk ratios for the association between hyperuricemia and new-onset CKD in non-CKD populations during follow-up. Results of summary analysis of these six studies showed a significant association between hyperuricemia and increasing risk of the incidence of CKD (summary OR, 2.59; 95% CI, 2.14–3.13) in a random effects model. Moderate heterogeneity was found among studies (*Q* = 9.55; *P* = 0.09; *I*^2^ = 61.2%) (Figure 4).

Results of meta-regression analysis indicated that heterogeneity was caused by follow-up year and sex proportion. One study from Japan only included male volunteers and had a follow-up of 18 years and reported a comparatively higher OR and contributed 44% of heterogeneity [20]. After excluding this study, no significant heterogeneity across the remaining studies was detected (*Q* = 0.480, *P* = 0.31, *I*^2^ = 17%). For these five other studies, the association between hyperuricemia and the onset of CKD was still significantly positive (summary OR, 2.11; 95% CI, 1.70–2.61).

### Subgroup analysis

Results of subgroup analysis according to sources of participants, geographical area, sex and follow-up year are shown in Table 2.

**Table 2:** Summary of relative risks for associations between SUA and development of CKD

**Subgroup**	**Reference**	**SOR**	**Tests for heterogeneity**		
		**(95% CI)**	***Q***	***P***	***I***^**2 **^**(%)**
**Geographic region**					
Asia	[17,19,23,25]	1.05 (1.03-1.08)^△^	30.7	<0.001*	90
Asia^#^	[19,23,25]	1.04 (1.01-1.06)^△^	1.76	0.42	0.00
Western	[18,26,27]	1.17 (1.11-1.22)^△^	0.64	0.724	0.00
**Population**					
Healthy people	[16,17,20,28]	2.59 (2.07-3.23)^▲^	6.08	0.02*	69
Diabetic	[18,24]	1.90 (1.30-2.78)^▲^	0.07	0.79	0
**Sex**					
Male	[21,22,26]	1.43 (1.05-1.94)^△^	32.98	0.001*	94
Female	[21,22,26]	1.21 (1.04-1.41)^△^	4.57	0.07	63
**Follow-up**					
<5 years	[2,19,23,25]	1.03 (1.01-1.06)^△^	0.19	0.66	0
	[16,17]	2.49 (1.79-3.46)^▲^	2.99	0.08	67
≥5 years	[17,18,26,27]	1.09 (1.04-1.14)^△^	2.63	0.45	0
	[18,20,24,28]	2.64 (2.09-3.32)^▲^	6.48	0.09	54

SOR: summary odds ratio; ^#^Studies in Asian areas (except study by Chang et al. [17]); ^△^OR calculated using continuous variables; per 1 mg/dL increase of uric acid levels; ^▲^OR calculated using dichotomous variables; hyperuricemia compared with normal; *heterogeneity exists.

For subgroup analysis for source of participants, no differences were observed in summary OR for associations between hyperuricemia and CKD in patients with diabetes and the community-based population (test for subgroup difference, *P* = 0.07). Both groups showed significant independent associations between hyperuricemia and new-onset CKD.

For geographical location, positive results were observed between SUA and the development of new-onset CKD in both Asian countries (summary OR, 1.05; 95% CI, 1.03–1.08) and Western countries (summary OR, 1.17; 95% CI, 1.11–1.22).

**Figure 5:**
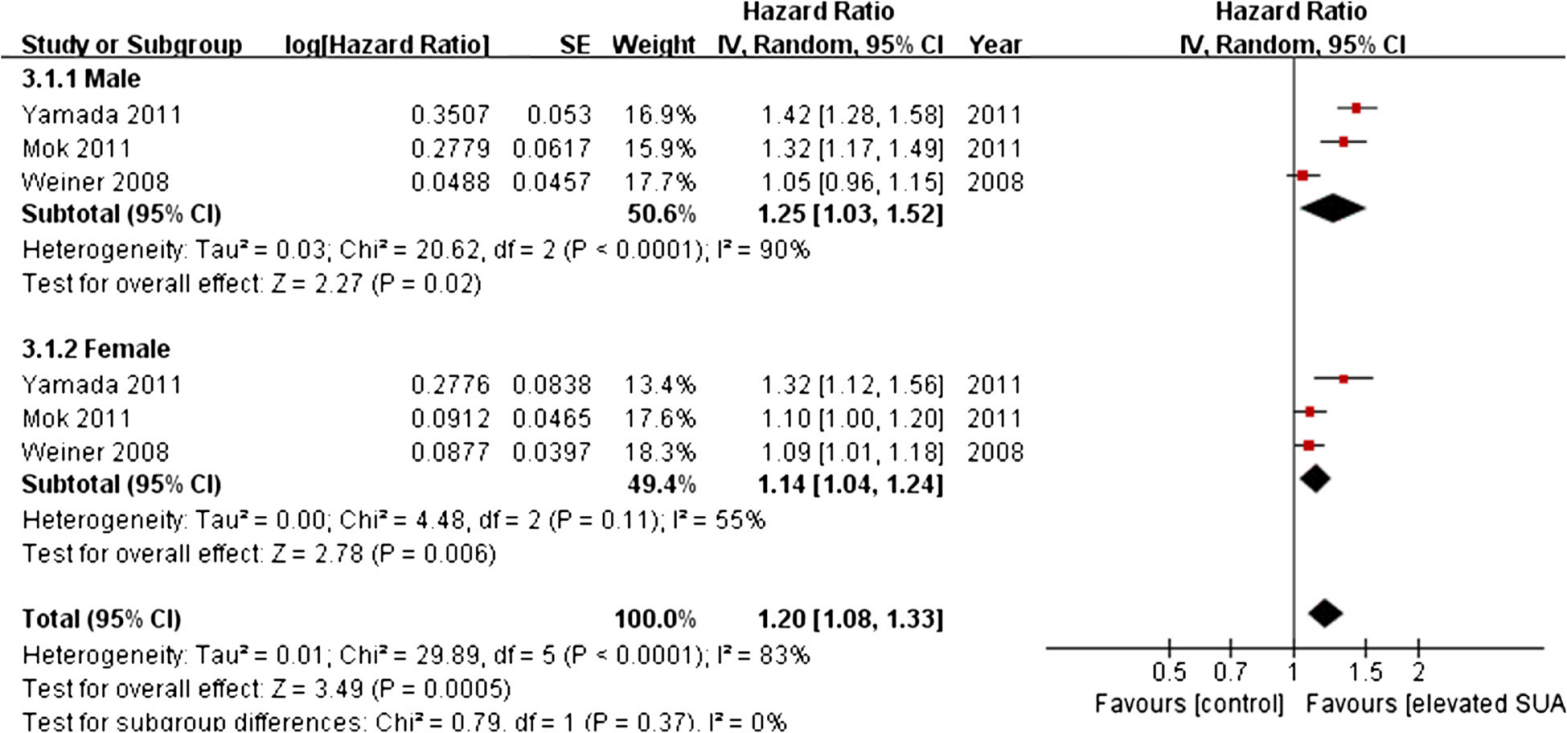
Forest plot of association between SUA and new-onset CKD in different genders.

Three studies provided results for males and females, and the results of pooled analysis by sex indicated a similar significant positive association between hyperuricemia and new-onset CKD in both males (summary OR, 1.43; 95% CI, 1.05–1.94.) and females (summary OR, 1.21; 95% CI, 1.04–1.41) (Figure 5). A study by Mok *et al.* reported that a stronger association beween hyperuricemia and CKD for in females than males, a finding contrary to results from the other studies, contributed to the heterogeneity of pooled analysis [21].

The subgroup of follow-up year revealed that the longer time period for observation, the greater the risk of the development of new-onset CKD in patients with elevated uric acid levels.

### Publication bias

**Figure 6:**
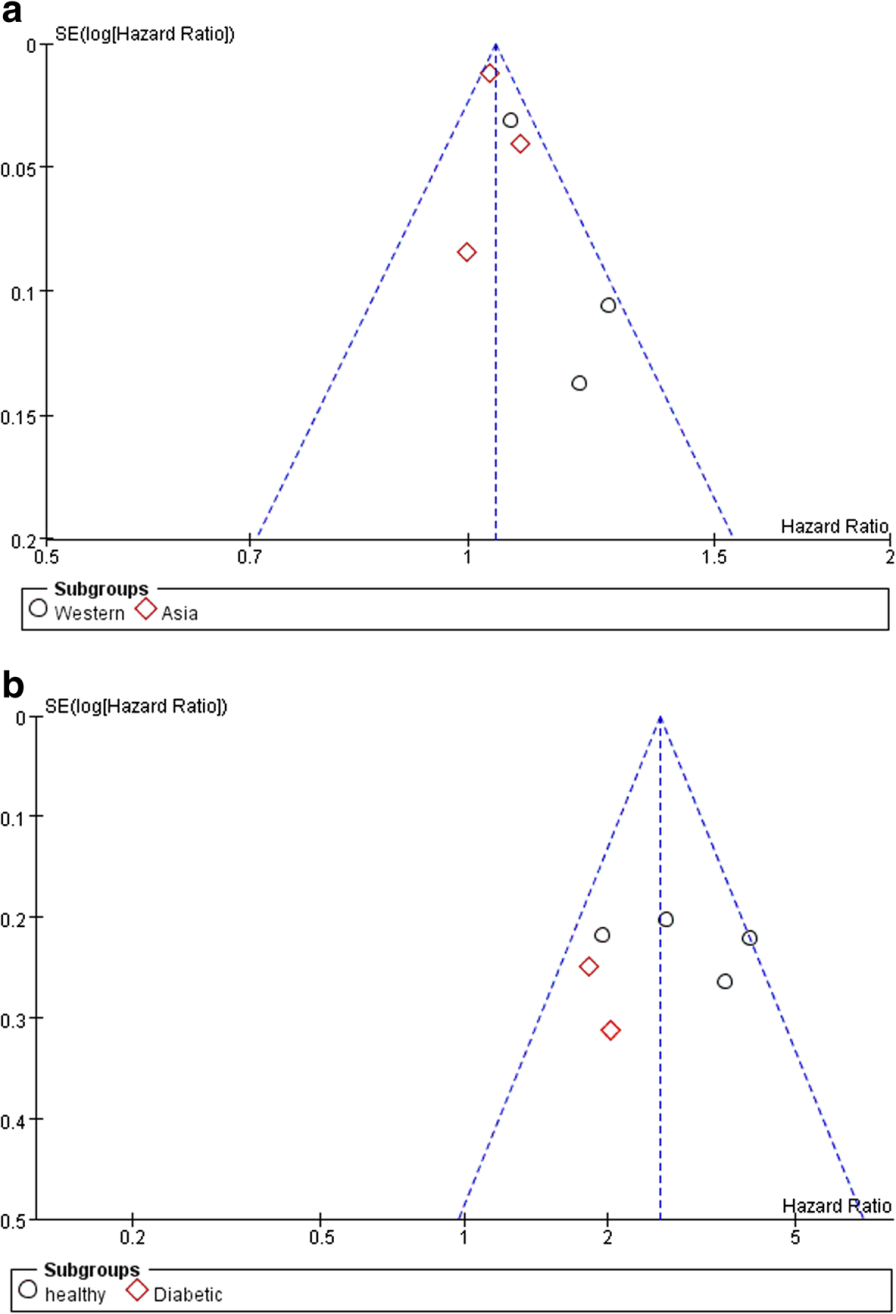
**Funnel plot of association between increasing SUA or hyperuricemia and new-onset CKD. a**. Funnel plot of association between increasing SUA and new-onset CKD, **b**. Funnel plot of association between hyperuricemia and new-onset CKD.

Funnel plots revealed no evidence of publication bias for associations between increasing SUA and new-onset CKD and the relationship between hyperuricemia and new-onset CKD (Figure 6a, Figure 6b). Results of the Begg adjusted rank correlation test for detecting publication bias were negative (SUA, *P* = 0.576; hyperuricemia, 0.452). The Egger regression asymmetry test showed no significance for either SUA (*P* = 0.176) or hyperuricemia (*P* = 0.977).

## Discussion

A previous meta-analysis conducted by Li *et al.* reported that uric acid level was independently associated with renal disease; however, the researchers included results from patients with end-stage renal disease or acute renal impairment and patients who received dialysis [29]. Additionally, the definition of renal dysfunction used by Li *et al.* did not follow the KDOQI CKD guidelines [12].

The present meta-analysis and systematic review found that elevated SUA was significantly positively associated with new-onset CKD in populations with normal renal function: individuals with hyperuricemia had an increased risk of new-onset CKD compared with those without hyperuricemia. Although the adjusted OR for the association between elevated SUA and the development of new-onset CKD was not great, it was found to be independent of sex, age, body mass index, alcohol intake, smoking, hypertension, metabolic syndrome, hypertriglyceridemia, diabetes and medication. In subgroup analysis, we found that the summary OR for the association between elevated SUA and development of new-onset CKD increased with increasing length of follow-up time, indicating hyperuricemia may play a role in the long-term progression of renal function. With regard to geographic distribution, subgroup analysis showed a stronger association between elevated SUA and CKD development in Western countries compared with Asian populations. This difference may be because of race, geographical or diet differences, as the Western diet might contain more purine-rich foods. Two studies by Chen and Xue *et al.* reported that the strength of the association between hyperuricemia and CKD was greater in males than in females [30,31]. However, the results of our subgroup analysis found that males and females had similar risks of CKD and new-onset CKD associated with hyperuricemia. High-quality etiological studies are therefore needed.

Our study is of significant clinical and public health importance. CKD is a very common disease and its prevalence is increasing in both developed and developing countries. Treating hyperuricemia may delay or even prevent the onset of CKD. Clinical studies conducted by Feig *et al.* suggested that treating hyperuricemia in adolescents with newly diagnosed hypertension was effective at lowering blood pressure, which indirectly decreased the risk of incidence of CKD [32]. A case–control study conducted by Kanbay *et al.* revealed that allopurinol intervention in individuals with eGFR >60 mL/min/1.73 m^2^ significantly increased eGFR at follow-up [33]. A recent meta-analysis by Bose *et al.* reported that uric acid-lowering therapy with allopurinol could retard the progression of CKD, but had no effect on proteinuria and blood pressure [34]. A significant association between metabolic syndrome and CKD has been reported in several clinical studies and in one meta-analysis [35].

The present meta-analysis has potential limitations. First, observational cohort studies may have unknown confounding attribution bias and selection bias, which may distort the true effects. Second, OR or HR calculated using continuous and dichotomous variables were pooled to reduce heterogeneity. Some heterogeneity caused by different statistical methods is inevitable; for example, heterogeneity for pooled analysis in males was caused by one study using uric acid per SD for their calculations. Third, confounding variables are likely to be present. Hyperuricemia may also reflect an unhealthy lifestyle, such as smoking, obesity and hyperlipidemia. These risk factors have been confirmed to play a pathogenic role in diabetes, hypertension and metabolic syndrome, which also contribute to renal dysfunction. Half the studies examined did not make adjustments for metabolic syndrome and diabetes in their calculations. Fourth, heterogeneity of cumulative analysis, which may be caused by population source, follow-up year and gender, could only be partially explained by subgroup analysis on examining these factors. Subgroup analysis by race and different stages of CKD was not possible because of a lack of data. Finally, as the publication bias test was statistical, it is possible for publication bias to exist as the meta-analysis can only use the published studies.

As with any observational study, associations do not imply causality. For example, in a recent Mendelian randomization analysis of two large cohorts, though estimates confirmed known observational associations between plasma uric acid and hyperuricemia with risk of ischemic heart disease, when using genotypic instruments for uric acid and hyperuricemia, the author saw no evidence for causal associations between uric acid and ischemic heart disease [36]. Similarly, the association found with our meta-analysis does not necessarily mean a causal relationship between SUA and CKD. However, our results were consistent with biologically plausible explanations mentioned in other studies.

Traditionally, gout is associated with renal disease while hyperuricemia results in intraluminal crystals in collecting ducts of nephrons [37]. Uric acid crystals adhere to the surface of renal epithelial cells and induce an acute inflammatory response [38]. Other than the uric acid crystal effect, hyperuricemia has been reported to cause an afferent renal arteriopathy and tubulointerstitial fibrosis in the kidney by activating the renin–angiotensin–aldosterone system [39]. It has also been shown that hyperuricemia activates cytoplasmic phospholipase A2 and inflammatory transcription factor nuclear factor kappa-β, which inhibit proximal tubular proliferation [40]. Furthermore, an elevated SUA level accelerates renal injury progression in the remnant kidney model via a mechanism linked to high systemic blood pressure and cyclooxygenase-2-mediated, thromboxane-induced vascular disease [41]. In rat experiments conducted by Ryu *et al.*, uric acid induced phenotypic transition of cultured renal tubular cells, as epithelial-to-mesenchymal transition, which is a known mechanism for renal fibrosis [42].

We found that hyperuricemia could be a risk factor for CKD. For patients with subclinical kidney disease, hyperuricemia can be the consequence of decreased renal uric acid excretion, which could in turn further exacerbate kidney function. Therefore, the causal relationship between hyperuricemia and CKD is far more complicated than a simple cause-and-effect relationship. This means that the results of the current study should be interpreted with caution.

Regardless of whether elevated SUA is solely a marker of subclinical chronic renal dysfunction or an independent risk factor for the development of CKD, careful attention is warranted. Our study emphasizes the need to identify individuals with hyperuricemia, as early intervention that decreases SUA levels may lower the risk of developing CKD. Randomized controlled studies of high quality are required to investigate the effects of decreasing uric acid levels in CKD patients and in individuals at high risk of developing CKD.

## Conclusions

This systematic review and meta-analysis demonstrated that, in the long-term follow-up of non-CKD individuals, elevated SUA levels showed an increased risk of developing chronic renal dysfunction.

## Abbreviations

CKD: Chronic kidney disease; KDOQI: National kidney foundation kidney disease outcomes and quality initiative; NOS: Newcastle–ottawa scale.

## Competing interests

The authors declare that they have no competing interests.

## Authors’ contributions

PF conceived the study and participated in its design. LL and CY participated in the design of the study and performed statistical analyses. The manuscript was prepared by LL, CY, YZ, XZ, FL and PF. All authors participated in discussions about the manuscript and approved the final version.

## Pre-publication history

The pre-publication history for this paper can be accessed here:

http://www.biomedcentral.com/1471-2369/15/122/prepub

## Supplementary Material

Additional file 1 The MOOSE Checklist of the article. Click here for file

Additional file 2 The NOS quality assessment of the included cohort studies. Click here for file
