# Supplementary material for: Is hyperuricemia an independent risk factor for new-onset chronic kidney disease?: a systematic review and meta-analysis based on observational cohort studies
Source: BMC Nephrol. 2014 Jul 27;15:122. doi: 10.1186/1471-2369-15-122 (PMC4132278; doi:10.1186/1471-2369-15-122)
Supplement: Additional file 1 — The MOOSE Checklist of the article. [file 1471-2369-15-122-S1.docx]

**The MOOSE Checklist of the article**

| **Criteria** | | **Brief description of how the criteria were handled in the meta-analysis** |
| --- | --- | --- |
| **Reporting of background should include** | |  |
| √ | Problem definition | Hyperuricaemia has been proved to be associated with CKD, however, whether an elevated uric acid level is an independent risk factor for CKD remains debatable. |
| √ | Hypothesis statement | Increasing evidence suggests that hyperuricaemia may be a pathogenic factor for the development of CKD rather than just a marker of decreased renal uric acid excretion. |
| √ | A statement of objectives that includes the study population, the condition of interest, the exposure or intervention, and the outcome(s) considered | Population: people without CKD at the beginning of the follow-up.  Condition of interest: no interest conflicting.  Outcome: development of CKD |
| **Reporting of search strategy should include** | |  |
| √ | Qualifications of searchers | The credentials of the two investigators LL and YC are indicated in the author list. |
| √ | Search strategy, including time period included in the synthesis and keywords | Medline Ovid/Medline (1948 to present), Pubmed/Medline, Embase, and ISI Web/Web PubMed from 1970 –September 2013, keywords list in the method part of article |
| √ | Databases and registries searched | Medline Ovid/Medline (1948 to present), Pubmed/Medline, Embase, and ISI Web/Web PubMed |
| √ | Search software used, name and version, including special features | We did not employ a search software. EndNote was used to merge retrieved citations and eliminate duplications |
| √ | Use of hand searching | We hand-searched bibliographies of retrieved papers for additional references, |
| √ | List of citations located and those excluded, including justifications | Details of the literature search process are outlined in the flow chart. The citation list is available upon request |
| √ | Method of addressing articles published in languages other than English | Only studies published in English were included in our meta-analysis. |
| √ | Method of handling abstracts and unpublished studies | Only published studies were included in our analysis. |
| √ | Description of any contact with authors | No contact with authors |
| **Reporting of methods should include** | |  |
| √ | Description of relevance or appropriateness of studies assembled for assessing the hypothesis to be tested | Detailed inclusion and exclusion criteria were described in the methods section. |
| √ | Rationale for the selection and coding of data | Data extracted from each of the studies were relevant to the population characteristics, study design, exposure, outcome, and possible confounding factors of the association. |
| √ | Assessment of confounding | All adjusted confounding factors were listed on Table 1. Studies reported more than three adjusted factors were included for pooled-analysis. |
| √ | Assessment of study quality, including blinding of quality assessors; stratification or regression on possible predictors of study results | Study quality were assessed by using New-Ottawa scale.  Meta-regression analysis and Sensitivity analysis were conducted. |
| √ | Assessment of heterogeneity | Heterogeneity of the studies were explored within two types of study designs using Cochrane’s Q test of heterogeneity and I^2^ statistic that provides the relative amount of variance of the summary effect due to the between-study heterogeneity. |
| √ | Description of statistical methods in sufficient detail to be replicated | Description of methods of meta-analyses, sensitivity analyses, meta-regression and assessment of publication bias are detailed in the methods. |
| √ | Provision of appropriate tables and graphics | We included 1 flow chart detailing the terms used for database search and study selection, ,2 forest plot of for association between SUA and CKD, hyperuricemia and CKD respectively, 1 table of subgroup analyses and two funnel plot for publication bias . |
| **Reporting of results should include** | |  |
| √ | Graph summarizing individual study estimates and overall estimate | Figure 2, 3 |
| √ | Table giving descriptive information for each study included | Table 1 |
| √ | Results of sensitivity testing | Detailed sensitive testing were described in results section |
| √ | Indication of statistical uncertainty of findings | 95% confidence intervals were presented with all summary estimates. Potential reasons for the observed heterogeneity was discussed. |
| **Reporting of discussion should include** | |  |
| √ | Quantitative assessment of bias | Sensitivity analyses indicate heterogeneity in strengths of the association due to most common biases in observational studies. |
| √ | Justification for exclusion | The exclusion criteria were described in method section. The process of exclusion were listed on Figure 1. |
| √ | Assessment of quality of included studies | Table 2 |
| **Reporting of conclusions should include** | |  |
| √ | Consideration of alternative explanations for observed results | For patients with subclinical kidney disease, hyperuricemia can be the consequence of decreased renal uric acid excretion, which could in turn further exacerbate kidney function. Therefore, the causal relationship between hyperuricemia and CKD is far more complicated than a simple cause-and-effect relationship. |
| √ | Generalization of the conclusions | In the long-term follow-up of non-CKD individuals, elevated SUA levels showed an increased risk of developing chronic renal dysfunction. |
| √ | Guidelines for future research | We recommend future randomized studies on the role of urate-lowing drug for preventing CKD. |
| √ | Disclosure of funding source | No separate funding was necessary for the undertaking of this systematic review. |
