# Supplementary material for: Is hyperuricemia an independent risk factor for new-onset chronic kidney disease?: a systematic review and meta-analysis based on observational cohort studies
Source: BMC Nephrol. 2014 Jul 27;15:122. doi: 10.1186/1471-2369-15-122 (PMC4132278; doi:10.1186/1471-2369-15-122)
Supplement: Additional file 2 — The NOS quality assessment of the included cohort studies. [file 1471-2369-15-122-S2.doc]

Table. The NOS quality assessment of the included cohort studies

| Study | Representativeness of the exposed cohort | Selection  of the non exposed cohort | Ascertainment of exposure | Outcome of interest not present at start of study | Comparability of cohorts | Assessment of outcome | follow-up long enough | Adequacy of follow up | Score |
| --- | --- | --- | --- | --- | --- | --- | --- | --- | --- |
| **Ryoo et al.**  **2013** | YES | YES | YES | YES | YES | YES | YES | NO | 7 |
| **Chang et al.**  **2013** | YES | YES | YES | YES | YES | NO | YES | NO | 6 |
| **Zoppini et al.**  **2012** | YES | NO | YES | YES | YES | YES | YES | NO | 6 |
| **Sonoda et al.**  **2011** | YES | YES | YES | YES | YES | YES | NO | NO | 6 |
| **Kawashima et al.**  **2011** | YES | YES | NO | NO | NO | YES | YES | NO | 4 |
| **Mok et al.**  **2011** | YES | YES | YES | YES | YES | YES | YES | NO | 7 |
| **Yamada et al.**  **2011** | YES | NO | YES | YES | YES | YES | NO | NO | 5 |
| **Wang et al.**  **2011** | YES | NO | YES | YES | YES | YES | NO | NO | 5 |
| **Jalal et al.**  **2010** | NO | NO | NO | YES | YES | NO | YES | NO | 3 |
| **Yen et al.**  **2009** | NO | YES | YES | NO | YES | YES | YES | NO | 5 |
| **Weiner et al.**  **2008** | YES | YES | YES | YES | YES | YES | YES | NO | 7 |
| **Obermayr et al.**  **2008** | NO | YES | YES | YES | YES | YES | YES | YES | 7 |
| **Domrongkitchaiporn et al. 2005** | YES | NO | YES | NO | YES | YES | YES | YES | 6 |

YES: the study meet the item in the scale; NO: the study didn’t mentioned it(unclear) or failed to meet that item.
